# Ti_3_C_2_T_x_ MXene-Supported Anisotropic Crystallisation of Solvent-Casted p(VDF-TrFE) Films for Improved Elastic Deformation and Piezoelectric Response

**DOI:** 10.3390/polym18202488

**Published:** 2026-10-12

**Authors:** Vanja Kokol, Branko Nečemer, Eugenio Gibertini, Vid Bobnar

**Affiliations:** 1 Faculty of Mechanical Engineering, University of Maribor, 2000 Maribor, Slovenia; branko.necemer@um.si; 2 Department of Chemistry, Polytechnic University of Milan, 20133 Milan, Italy; eugenio.gibertini@polimi.it; 3 Department of Condensed Matter Physics, Jožef Stefan Institute, 1000 Ljubljana, Slovenia; vid.bobnar@ijs.si

**Keywords:** p(VDF-TrFE), Ti_3_C_2_T_x_ MXene, β-phase formation, mechanical deformation, dielectric behaviour, piezoelectric response

## Abstract

This study investigates the percolation limit of solvent-casted p(VDF-TrFE) film through its processing (thermal treatment) and the addition of 2D Ti_3_C_2_T_x_ MXene, by evaluating their distribution using SEM-EDX and determining the electroactive crystalline phases using FTIR and XRD spectroscopies, while evaluating the film’s dielectric permittivity and piezoelectric response after a mechanical deformation cycle (elongation). The robust interactions of the -CH_2_/-CF_2_ groups of copolymer (55/45 mol%) with the terminal groups (-OH/F) of MXene influence their distributions and alignment along the MXene nanosheets, and, consequently, the higher and anisotropically distributed β-phase crystallinity near its percolation limit (0.0015 *w*/*w*% of loading). This exhibited a piezoelectric behaviour of d33 ~19 pC/N after e-polishing and ~24 pC/N after 50% of elongation performed at an ambient temperature. Such an interconnected and uniform film achieves a dielectric permittivity of ~22, exhibits intrinsic flexibility (fracture resistance), and facilitates effective load transfer during elastic deformation while maintaining the piezoelectric response, which could not be achieved for a pure p(VDF-TrFE) film.

## 1. Introduction

Due to their great potential for application in flexible electronics (robotics and healthcare sensors) or self-powering devices to be integrated directly with wearable electronics, poly(vinylidene fluoride) and its copolymers have high attention, being related to their moderate and tunable piezoelectric and dielectric properties, as well as high flexibility and good processability with easy preparation by different morphologies (as fibres, thin films and porous foams), and by various technologies (such as screen or inkjet printing, solution casting, hot pressing, electrospinning, spin-coating, etc.) [1]. Only piezoelectric sensors offer many advantages over other pressure-type sensors, due to their high sensitivity, wide temperature/pressure measurement range, self-powered capability and low power consumption [2]. As passive sensors they utilise the piezoelectric effect to convert external mechanical energy into charge, thus realising different sensing functions (pressure, vibration, motion, tactile).

Poly(vinylidene fluoride-co-trifluoroethylene) (p(VDF-TrFE)) is one of the most important piezoelectric copolymers, because it combines ferroelectricity, mechanical flexibility, low density, chemical stability and moderate piezoelectric response (d_33_ ≈ 20–30 pC/N; [3]). Compared with neat PVDF, the TrFE comonomer stabilises the electroactive all-trans (β-phase), allowing strong dipole alignment and piezoelectric response without the extensive mechanical stretching usually required for PVDF. The piezoelectric response of p(VDF-TrFE) is thus governed mainly by β-phase crystallinity, dipole orientation, ferroelectric domain switching, annealing conditions, film morphology and poling efficiency. Many techniques have been proven to induce dipole alignment for increasing the proportion of the crystalline *β* phase, such as mechanical stretching [2], tuning of the solvent composition [4], and blending with piezoceramics (such as BaTiO_3_, ZnO, CoFe_2_O_4_) [5,6], or conductive and semiconductive nanofillers (such as Ag nanomaterials, graphene derivatives, MXenes, carbon nanotubes, etc.) [7,8,9,10,11]. The nanoceramics provide nucleation sites via favourable interactions between the p(VDF-TrFE) polymer chains, thereby enhancing performance through several mechanisms [12], including an increased β-phase and crystallinity, improved interfacial polarisation, local electric-field concentration during poling, mechanical reinforcement, and direct contributions from their piezoelectricity. BaTiO_3_ thus yielded an even d_33_ of ∼33 pC/N at a very high loading (44 wt%) and using two-pole steps [13]. However, excessive piezo-filler loading can cause aggregation, poor dispersion, electrical leakage, reduced flexibility and deterioration of the piezoelectric response. On the other hand, conductive fillers not only enhance the structure of the β-phase and increase the electric field intensity (leading to increased polarisation), but also modify the charge transport, interfacial polarisation and the local electric field distribution. However, these fillers can improve sensitivity and energy-harvesting output only at low or optimised concentrations, whereas high loadings may increase the dielectric losses or cause short-circuiting during poling, thereby increasing conductivity and the leakage current [14]. Semiconductive 2D fillers seem to be among the most effective among them for enhancing p(VDF-TrFE) dielectric and piezoelectric performance, because low-to-moderate filler loading can bring stronger interfacial bonding at homogeneous nanosheet dispersion, and thus induce β-phase formation, enlarge lamellae dimensions and align micro-dipoles [15,16].

Achieving uniform filler dispersion, preventing aggregation and leakage, and maintaining mechanical flexibility and deformability while improving long-term piezo stability are, however, still major challenges. Pure p(VDF-TrFE) films are intrinsically flexible, due to their semicrystalline polymer structure and relatively low elastic modulus compared with ceramic piezoelectrics. Under elastic deformation, polymer-chain rotation, dipole reorientation and reversible crystalline deformation contribute to a stable electromechanical response. The introduction of 2D nanosheets into such a polymer matrix can either improve or worsen these properties, depending on the lateral size and concentration of the filler, the quality of the dispersion, interfacial interactions and the morphology of the film. The latest research on elastic deformation thus focuses on how these composites respond under tensile, bending, cyclic and dynamic mechanical loading while maintaining piezoelectric and dielectric functionality [17,18]. The state of the art indicates that the suitable incorporation of 2D filler can, simultaneously, reinforce the polymer matrix and preserve mechanical flexibility when the filler concentration and interface structure are optimised carefully [19], thereby achieving synergy between the filler and the matrix.

In this study, micro-sized 2D Ti_3_C_2_T_x_ MXene nanosheets were used as nucleating agents to increase both the electroactive (β-phase formation) crystallinity of the p(VDF-TrFE) and improve stress transfer within the composite under mechanical deformation of solvent-casted and thermally annealed nanocomposite films. The interactions between the p(VDF-TrFE) and MXene, their distribution and orientation, as well as their influence on the polymorphic phase formation and crystallinity of p(VDF-TrFE), were evaluated by SEM-EDX, FTIR, and XRD spectroscopies. In addition to the dielectric behaviour and piezoelectric response, the effects were also investigated on their mechanical stability and deformation under uniaxial tensile testing.

## 2. Materials and Methods

### 2.1. Materials

The p(VDF-TrFE) (55/45 mol%, FP45) powder was purchased from Arkema Piezotech S.A.S., Pierre-Bénite Cedex, France. Fast-drying (~10 min at 20 °C) Leitsilber 200 conductive silver ink (45% Ag content of ~16 µm grain size) of 0.02–0.04 ohms/sq was obtained from Ted Pella, Inc., Redding, CA, USA. The methyl ethyl ketone (MEK), hydrofluoric acid, HCl, LiCl, and ethanol were purchased from Merck KGaA, Darmstadt, Germany.

### 2.2. Preparation of Ti_3_C_2_T_x_ MXene Nanosheets and Their Dispersion in MEK

The Ti_3_C_2_T_x_ MXenes were synthesised through the HCl/HF mixed-acid approach. A total of 10 g of Ti_3_AlC_2_ MAX phase (purchased from Laizhou Kai Kai Ceramic Materials Co., Ltd., Laizhou, Shandong Province, China) was added slowly to an acidic mixture of 30 mL of hydrofluoric acid (40 wt%), 120 mL of HCl (37 wt%) and 60 mL of H_2_O and stirred for 24 h at 35 °C. The multilayered Ti_3_C_2_T_x_ was washed by centrifugation multiple times (3500 rpm, 5 min, 50 mL vial) until a pH >5.5 was achieved, and subsequently centrifuged 3 times with ethanol to obtain a high-concentration stock suspension. To prepare fully delaminated MXenes, an appropriate amount of stock suspension, corresponding to 2 g of solid multilayered Ti_3_C_2_T_x_, was centrifuged, washed with water, and stirred for 24 h in a solution containing 2 g LiCl in 40 mL of H_2_O to intercalate Li+ ions. Finally, delamination was achieved by first performing multiple centrifugations (3500 rpm, 5 min), followed by vortexing the vial for 30 min at 2400 rpm [20], thereby also purifying it from LiCl. The MXene suspension in MEK was obtained by solvent exchange via several centrifugation and washing cycles with MEK (3000–4000 rpm, 10 min), which was stored at 4–6 °C. The stable supernatant had a final MXene concentration of 0.05 wt%. The dynamic light scattering measurement (conducted on an Anton Paar Litesizer DLS 500, Graz, Austria) of 0.005 wt% MXene suspended in MEK showed a relatively low polydispersity (~25%) in the size distribution (~3.5 µm) of the nanosheets, confirming that the MXene preparation in MEK was appropriate. Although DLS measurements for 2D materials are methodologically flawed, the obtained hydrodynamic radius (~4–8 µm) of the MXene is quite consistent with the lateral size obtained by SEM imaging, as shown in Figure S1.

### 2.3. Fabrication of Nanocomposite Films

P(VDF-TrFE) films with and without different Ti_3_C_2_T*_x_* MXene content were prepared by the solution casting method, as shown in Table 1. For that purpose, p(VDF-TrFE) was first dissolved in MEK under continuous magnetic stirring at 60 °C for a few hours, to obtain a solution with 7 wt% concentration. The 0.05 wt% MXene solution dispersed in MEK was vortexed for at least 5 min before being added in various volumes (0.7–17.5 mL) to the p(VDF-TrFE) solution, with a further 3 min vortexing at 1500 rpm. The prepared solutions were cast (10 mL) onto a glass Petri dish with a diameter of 90 mm, dried overnight at room temperature, and then for a few days at 50 °C to form uniform films. After solvent evaporation the formed films were peeled off the Petri dish and annealed at 120 °C or 145 °C in an oven (Muffle furnace Nabertherm L3/11/B170, Nabertherm GmbH, Lilienthal, Germany) for 60 min, then allowed to cool to room temperature.

The as-prepared film samples (shown in Figure S2) have the same thickness (47–56 μm) as when measured using a Kafer Prec Micrometer 0.001 mm F 1000/30 (Käfer Messuhrenfabrik GmbH & Co. KG, Villingen-Schwenningen, Germany). After the electrode configuration (about ~100 nm thick silver conductive ink with a diameter of 4 mm applied to the upper and bottom surfaces), the samples were polarised at room temperature to align the p(VDF-TrFE) dipole moments. The samples were immersed in silicone oil for a few min to ensure a uniform coating. Subsequently, an external DC electric field was applied, and increased from 0 to 5000 V at a rate of 500 V min^−1^. The maximum voltage was then maintained for an additional 10 min. Such prepared samples were used for dielectric, piezoelectric and mechanical testing. It should be emphasised that the polarisation in silicone oil prevents electrical breakdown in the experimental setup, as the breakdown voltage of the oil is higher than that of air. By measuring the dielectric response of both the raw and oil-soaked samples prior to polarisation, it was confirmed that the oil neither affects the material nor is absorbed by it.

### 2.4. Characterisation

#### 2.4.1. Scanning Electron Microscopy (SEM) and Energy-Dispersive X-Ray (EDX) Spectroscopy

SEM imaging was performed by using a low-vacuum scanning electron microscope, FEI Quanta 200 3D (Thermo Fisher Scientific Inc., Waltham, MA, USA), equipped with an EDX spectrometer (Inca 350, Oxford Instruments Nanoanalysis Ltd., High Wycombe, UK) for micro-chemical mapping. The film surfaces and cross-sections were analysed to get information on the morphological features and MXene dispersion. The MXene suspension was droplet-applied onto an SEM stub holder with graphite tape, followed by drying, before being examined for SEM-EDX imaging.

#### 2.4.2. X-Ray Diffraction (XRD) Spectroscopy

The XRD spectra of the film samples were recorded in the angular range 2Θ from 5 to 80° with a step size of 0.04° and a collection time of 5 s/step by using a diffractometer with CuK*α* radiation and a Sol-X energy-dispersive detector (D4 Endeavor, Bruker AXS, Karlsruhe, Germany).

#### 2.4.3. Fourier-Transform InfraRed (FTIR) Spectroscopy

The FTIR spectra were obtained using a Spectrum One FTIR spectrometer (Perkin–Elmer Inc., Springfield, IL, USA) with a Golden Gate ATR attachment and a diamond crystal. The absorbance spectra were collected by taking an average of 128 individual scans at a resolution of 1 cm^−1^, between 600 cm^−1^ and 4000 cm^−1^. The Spectrum 5.0.2/10 software programme was applied for the data analysis. All the measurements were carried out in duplicate.

#### 2.4.4. Dielectric Characterisation

The complex linear dielectric constant ε*(*ω*,*T*) = *ε*′ − *iε*″ was measured by a Novocontrol Alpha Analyser (Novocontrol Technologies GmbH & CO. KG, Montabaur, Germany). The amplitude of the probing ac electric signal was 1 V. The values of the real part of the dielectric constant, i.e., the dielectric permittivity, *ε*′, and the values of the real part of the electrical conductivity, *σ*′, were calculated from the actual measured quantities, capacitance *C* and electrical resistivity *R* via *ε*′ = *Cd*/(*ε*_0_*S*) and *σ*′ = *d*/(*RS*), where *d* is the sample thickness, *S* is the electrode area, and *ε*_0_ = 8.85 × 10^−12^ As/(Vm) is the permittivity of the free space. The imaginary part of the dielectric constant *ε*″ is connected to the electrical conductivity via *σ*′ = 2*πνε*_0_*ε*″. The dielectric response was detected as a function of frequency at room temperature.

#### 2.4.5. Piezoelectric Response

The macroscopic piezoelectric coefficient, d_33_, was determined using a commercial piezometer (PiezoMeter System PM300, Piezotest Pte. Ltd., London, UK) based on the Berlincourt (quasi-static) method. In this technique, a small oscillating force (2 N at 110 Hz in the present study) is applied along the normal direction, while the electrical response of the sample is processed and compared with an internal reference, enabling a direct reading of the d_33_ coefficient.

#### 2.4.6. Mechanical Analysis

The mechanical properties (engineering stress–strain behaviour) of the prepared films were evaluated by uniaxial tensile testing using a Zwick/Roell LTM 3 universal testing machine (ZwickRoell GmbH & Co. KG, Ulm, Germany). Rectangular specimens with approximate dimensions of 10 mm in width and 30 mm in length were cut from the films. The tensile tests were performed at room temperature using a constant crosshead speed of 2 mm/min, after applying a preload of 0.25 N to ensure proper specimen alignment and eliminate slack. The engineering stress was calculated by dividing the applied force by the initial cross-sectional area, while the engineering strain was determined from the crosshead displacement relative to the initial gauge length. At least two specimens from each film were tested to average a representative stress–strain curve, which was used subsequently to determine and evaluate all the reported mechanical properties, i.e., the yield stress and the corresponding yield strain. The yield point was defined as the first pronounced local maximum in the engineering stress, corresponding to the first point at which the stress reached a maximum before decreasing. To minimise the influence of experimental noise, only data points beyond the initial deformation region and within the material’s load-bearing region were considered in the peak detection procedure.

The cyclic tensile tests were performed using the same specimen geometry and testing conditions as those employed in the uniaxial tensile tests. Three independent tests were conducted using selected specimens, with each specimen subjected to a single loading–unloading cycle consisting of loading to 50% engineering strain followed by unloading to 0% strain. The film’s mechanical response was recorded.

## 3. Results and Discussion

### 3.1. Effect of MXene Addition on Film Thickness and Morphology

**Figure 1:**
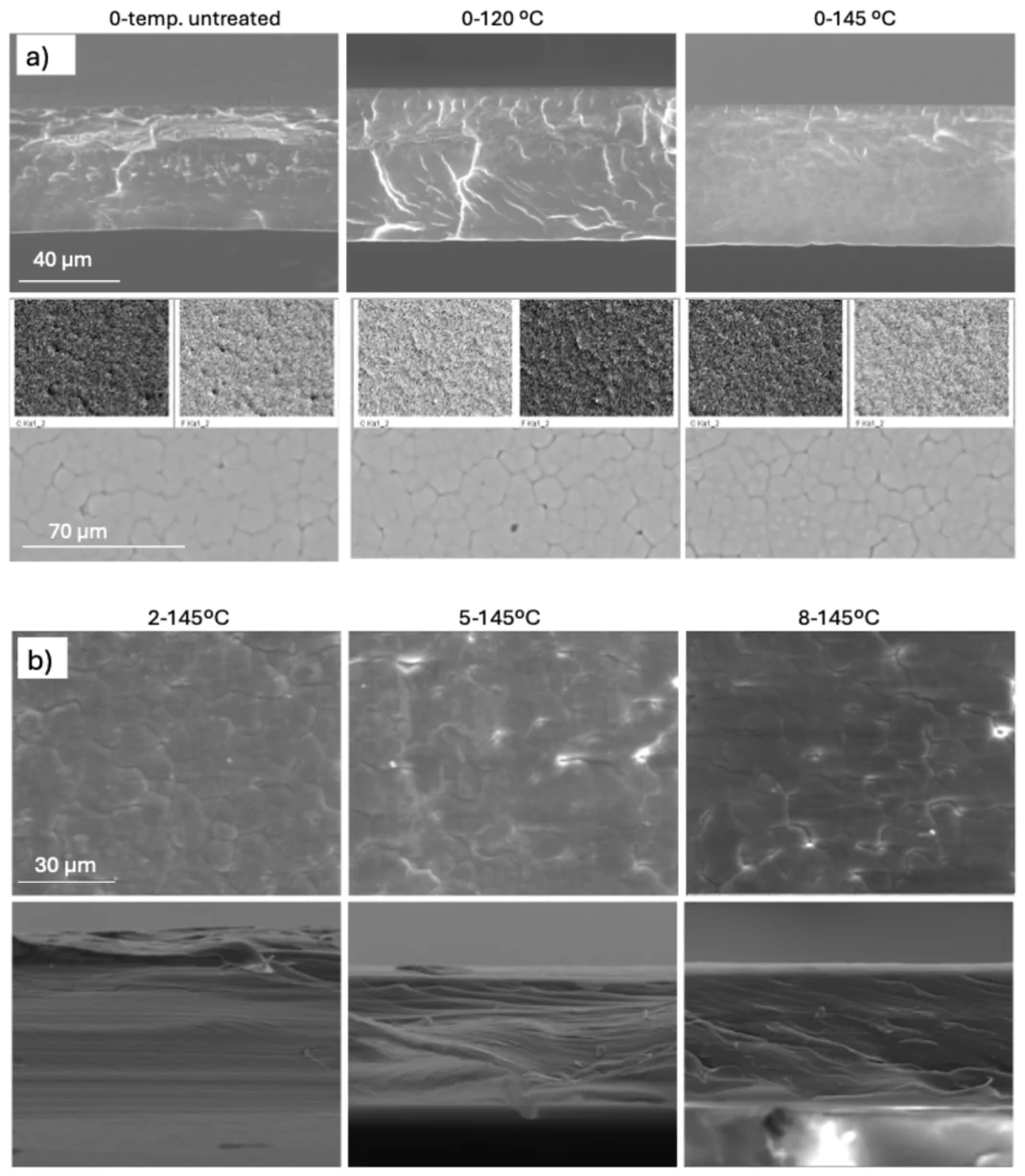
Surface topography and cross-section morphology of solvent-casted p(VDF-TrFE) films (**a**) before and after annealing at 120 °C and 145 °C, (**b**) and with different MXene additions after annealing at 145 °C, as obtained by SEM imaging.

The thickness of the films made with different Ti_3_C_2_T_x_ MXene contents and annealed at different temperatures showed a slight variation in film thickness, ranging between 47 and 56 μm, which was due to the difference in the cast liquids instead of to their viscosity due to the addition of MXene nanosheets. The surface topography and cross-sectional morphological structure of temperature-differently annealed p(VDF-TrFE) films, as well as representative composite samples, were examined with SEM imaging (Figure 1), and the elemental compositions were analysed by EDX spectroscopy. As the annealing temperature of the pure p(VDF-TrFE) films increases (Figure 1a), the sample structure becomes more homogeneous and the grain/patter size smaller, with more voids on the surface due to the higher evaporation rate of the solvent, which can increase the porosity. The molecular chains are, thus, oriented preferentially parallel to the substrate in the lamellar crystals that are structured perpendicular to it.

**Figure 2:**
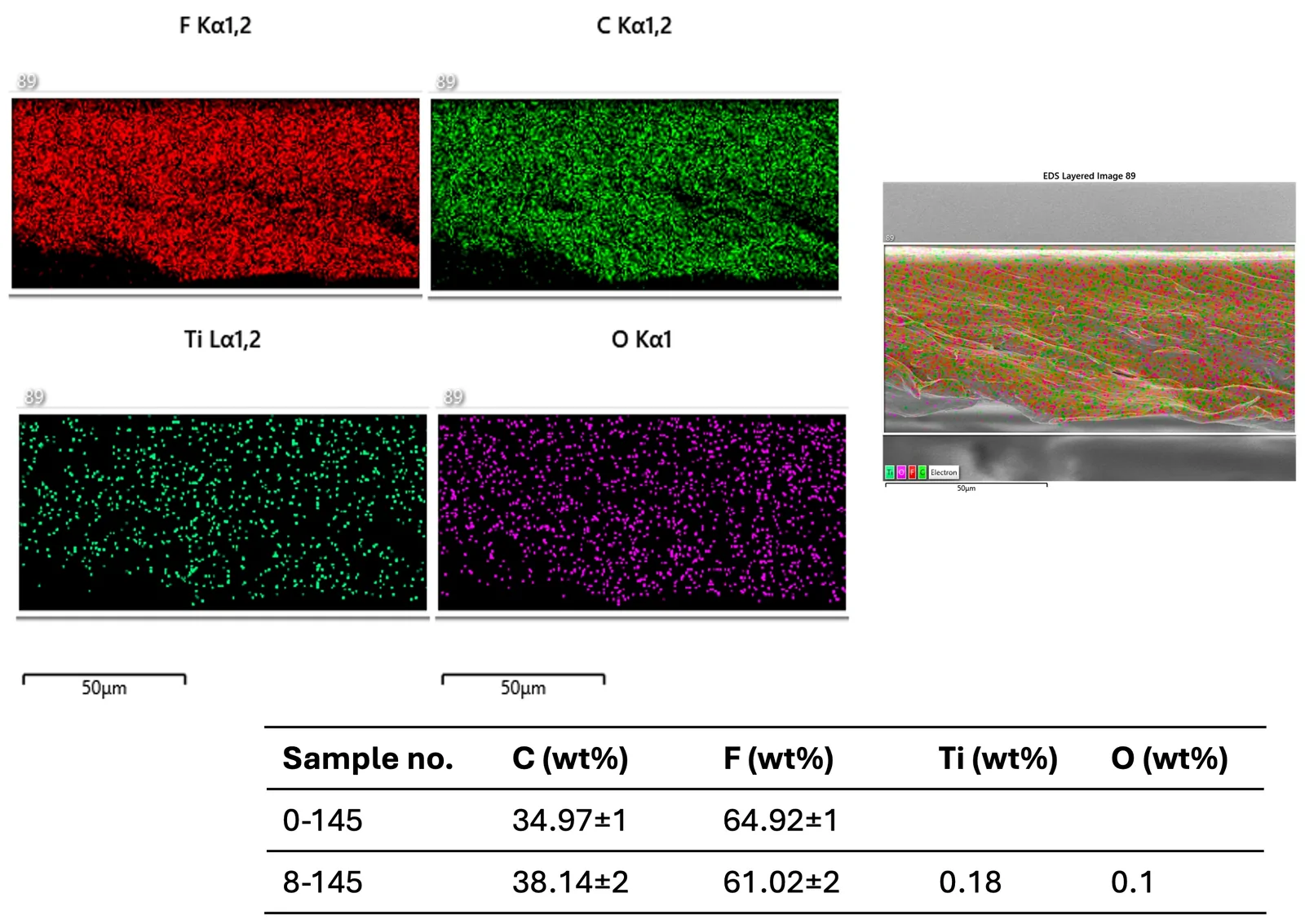
SEM-EDX images with elemental analysis (inserted Table) of solvent-casted p(VDF-TrFE) film with the highest Ti_3_C_2_T_x_ MXene addition after annealing at 145 °C.

The Ti_3_C_2_T_x_ MXene consists of a planar-like morphology of approximately 4–8 µm in diameter and single-to-few-layered nanosheets, as is clearly evident from the SEM image presented in Figure S1. The EDX spectra showed that most of the Al in the Ti_3_AlC_2_ MAX phase was removed after the HF etching and delamination steps, confirming the almost complete elimination of the Ti-Al bonds in the so-obtained Ti_3_C_2_T_x_ MXene. The spectra also indicated the presence of terminated (T_x_) groups (such as –F, =O, and –OH); some of them may also be present in oxide forms (TiO_2_ or Al_2_O_3_), because the samples were exposed to the laboratory atmosphere and subjected to vigorous ultrasonication and centrifugation during preparation. After the addition of such micron-sized Ti_3_C_2_T_x_ MXenes to p(VDF-TrFE), the morphology of the composite films developed a more planar shape with a smoother surface and larger grains/patterns, but still present porosity on the surface (Figure 1b). The local densities of such composite films were also getting more packed than those adjacent to the pure film, as the MXene possesses an increased surface roughness due to the presence of terminated groups, thus inducing steric effects in the p(VDF-TrFE) copolymer chains by robust interactions between the –OH/–F groups at the MXene nanosheets and –CF_2_/–CH_2_ groups at the p(VDF-TrFE) chains. However, although the elemental distribution of such a small quantity of MXene in the film structure could not be detected by EDX mapping (except for the film with the highest MXene content, Figure 2), their homogeneous dispersion throughout the polymeric structure can be assumed, as no distinctly clear or agglomerated areas were observed. The distribution of the Ti and F atoms in this composite film also confirmed the integration of Ti_3_C_2_T_x_ MXene in the p(VDF-TrFE) network by the appearance of Ti (and O), a slight increase in C, and a reduction in the F content. Consequently, it was expected that such preferentially aligned MXene nanosheets, although still within the randomly distributed polymer chains, would enhance the formation of electroactive β crystals, leading to the formation of intrinsic self-aligned dipoles within the p(VDF-TrFE) film and a better piezoelectric response.

### 3.2. Effect of MXene on Film Phase-Crystallinity Formations

In addition to the morphological features, the presence of MXene nanosheets also affects the crystalline structure of the p(VDF-TrFE) film. The p(VDF-TrFE) provides three types of polymorphic phases (the non-polar α-phase, semi-polar γ-phase and highly polar β-phase), among which only the β-phase is ferroelectric, with a large spontaneous polarisation along the axis; thus, it favours the alignment of electrical dipoles, exhibiting the major contribution in the piezoelectric response [21]. In such an arrangement, due to repulsion between the H atoms and adjacent highly electronegative F atoms, the –CF_2_ and –CH_2_ groups on the molecular chains of p(VDF-TrFE) are oriented on the same side of the carbon chains, thereby increasing the dipole moment and enhancing polarity. On the other hand, the specific orientation of these two groups thus also controls the formation of polymorphic phases during the crystallisation of the PVDF-TrFE polymers. The γ-phase is a stable intermediate state between the α-phase and the β-phase; hence, the distribution of phase fractions can provide insight into changes in the local electroactivity of the p(VDF-TrFE) film upon the addition of Ti_3_C_2_T_x_ MXene nanosheets, which was characterised by FTIR and XRD spectroscopies as well-established methods [7,10,11].

**Figure 3:**
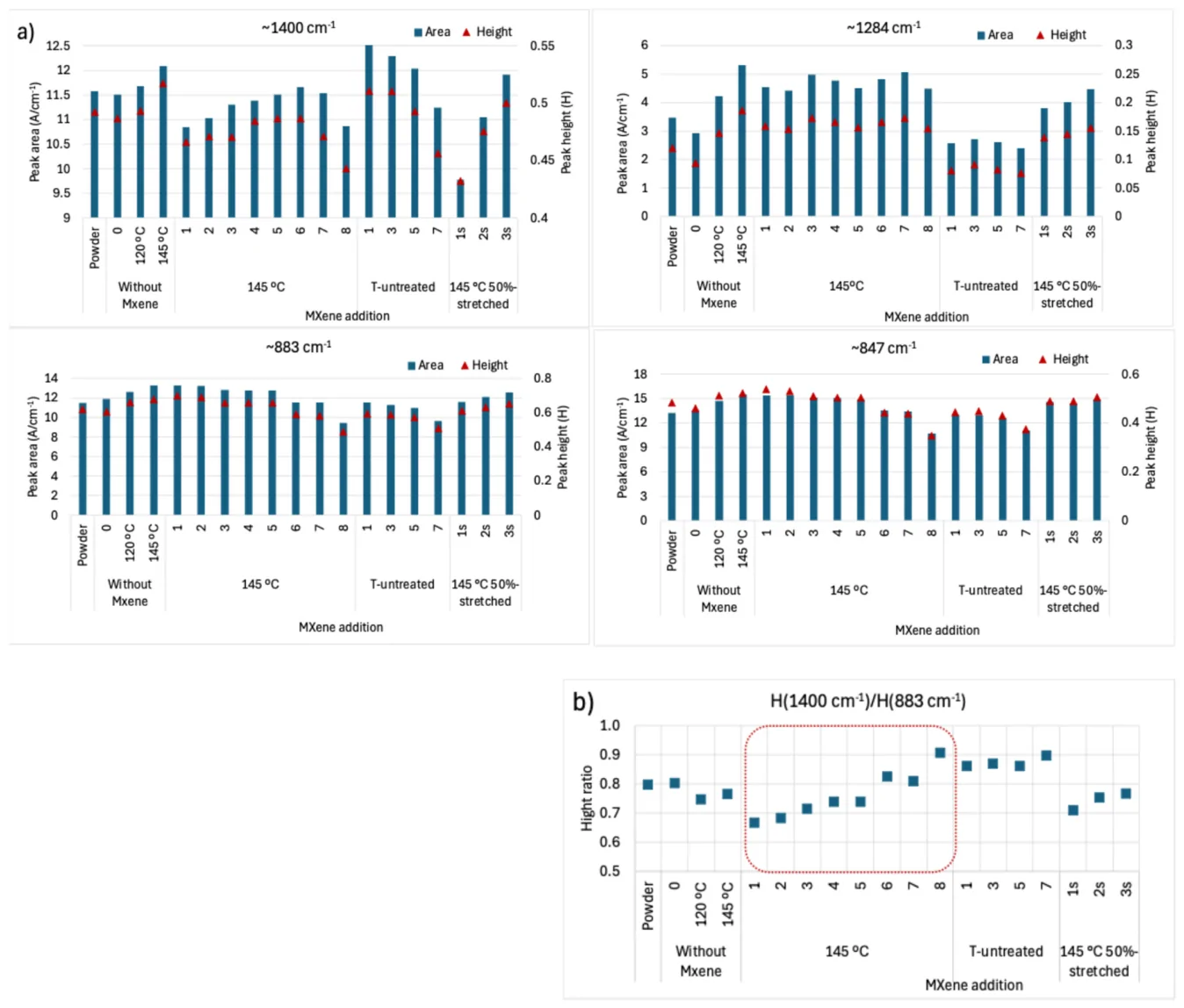
(**a**) Relative areas and heights of specific FTIR peaks, with (**b**) high (H) ratio values for chain orientation and absolute β-phase peaks, for differently prepared films, before and after 50% uniaxial elastic deformation (stretching, s) at room temperature.

The evolution of the FTIR spectra after each processing step is shown in Figure S3a. The spectra of the p(VDF-TrfE) film showed typical bands at ~847 cm^−1^ and ~883 cm^−1^ (associated with the CH_2_ rocking vibration and CF_2_ asymmetric stretching, respectively), the band at ~1284 cm^−1^ (attributed to out-of-phase/trans symmetric –CF_2_ stretching vibrations), the band at ~1174 cm^−1^ (antisymmetric –CF_2_ stretching vibrations for the trans sequence of one monomer), and the band at ~1400 cm^−1^ (–CH_2_ wagging vibrations specific to the polar phase). These four peaks can be used to evaluate the crystallinity of the films, and thus the presence of the piezoactive β-phase, as proposed in the literature [22,23,24]. In particular, an increase in peak at ~1284 cm^−1^ (associated with the ferroelectric/electroactive crystalline β-phase) and a doublet at 883–847 cm^−1^ (corresponding to the absolute/total electroactive phase consisting of β and γ phases) are expected, while the α-phase bands at ~975 and ~765 (assigned to CH_2_ twisting and CF_2_ skeletal bending, respectively) are expected to be reduced. Meanwhile, the intensity of the bands at ~1400 cm^−1^, assigned to CH_2_ coupled with CC, in which the transition moment is along the polymer chain direction (i.e., in-plane—along the substrate—orientation), has to increase. The peak heights and areas for all four corresponding phase peaks (shown in Figure 3a) followed this trend with increasing the annealing temperature, yielding the strongest absorbance peaks of crystalline phases (*trans*-zigzag sequences), as well as higher orientation of the chain (polar phase) for a post-annealed p(VDF-TrfE) film at 145 °C. The chain mobility, namely, increases as a function of temperature that favours the lowest energy conformation (from a randomly coiled chain state to an extended all *trans* state), thereby increasing its ferroelectric character after recrystallisation by cooling [25].

All the characteristic peaks for the p(VDF-TrfE) were still present for the composite films (Figure S3b), but some of them were decreased (in area and height) by increasing the MXene addition as compared to the pristine film, indicating the formation of differently structured phases and thus a crystalline structure. In addition, the peak intensity attributed to the α-phase (500–770 cm^−1^) was decreased, suggesting the low fraction of the α-phase in the bulk of the samples [26] due to the proximity to the MXene nanosheet surface. The spectra of the Ti_3_C_2_T_x_ MXene film had small shoulders at ~3400 cm^−1^, ~1000 cm^−1^, and more intense at ~600–500 cm^−1^, corresponding to the stretching vibrations of the –OH, C–F, and Ti–O bonds, respectively. These peaks cannot be distinguished in the spectrum of the composite films. In addition, all the characteristic peaks for the p(VDF-TrfE) containing the highest MXene content were shifting slightly, and some of them had obviously decreased in both intensity and area (above all, the doublet at ~883/847 cm^−1^) as compared to the reference film; however, it was still present in all the composite samples [27]. In the meantime, the peak area and intensity for the crystalline β-phase (at ~1284 cm^−1^) were still comparable to the film without MXene, confirming the presence of the electroactive phase. However, comparatively significantly higher intensities of the chain orientation-sensitive band at ~1400 cm^−1^ for the temperature-unannealed samples containing MXene—relative to the annealed ones—also indicate that the *trans*-zigzag polymer chains tend to orient preferentially in the substrate plane direction and parallel to the MXene surface, while acquiring more perpendicular orientation after annealing at 145 °C and increasing the crystalline β-phase; thus there are also more disordered structures when the MXene content is increased further [23]. At high MXene additions (from sample no. 6), the orientation of the chains namely decreased markedly (i.e., the intensity of the peak at ~1400 cm^−1^ is decreased) and their crystalline structure changed (which is reflected in a decrease in the intensity of the peaks ~883 cm^−1^ and ~847 cm^−1^), while the degree of crystallinity remained similar (i.e., the intensity of the peak at ~1284 cm^−1^ did not change). This indicates that the chains have lost part of their preferred orientation—perpendicular to the surface plane—at the expense of increased crystallinity, resulting in an even more isotropic or disordered structure. For such a structure it became more difficult for the –CF dipoles to switch along during the poling direction, and hence might influence their polarisation and, consequently, reduce the piezo-response.

**Figure 4:**
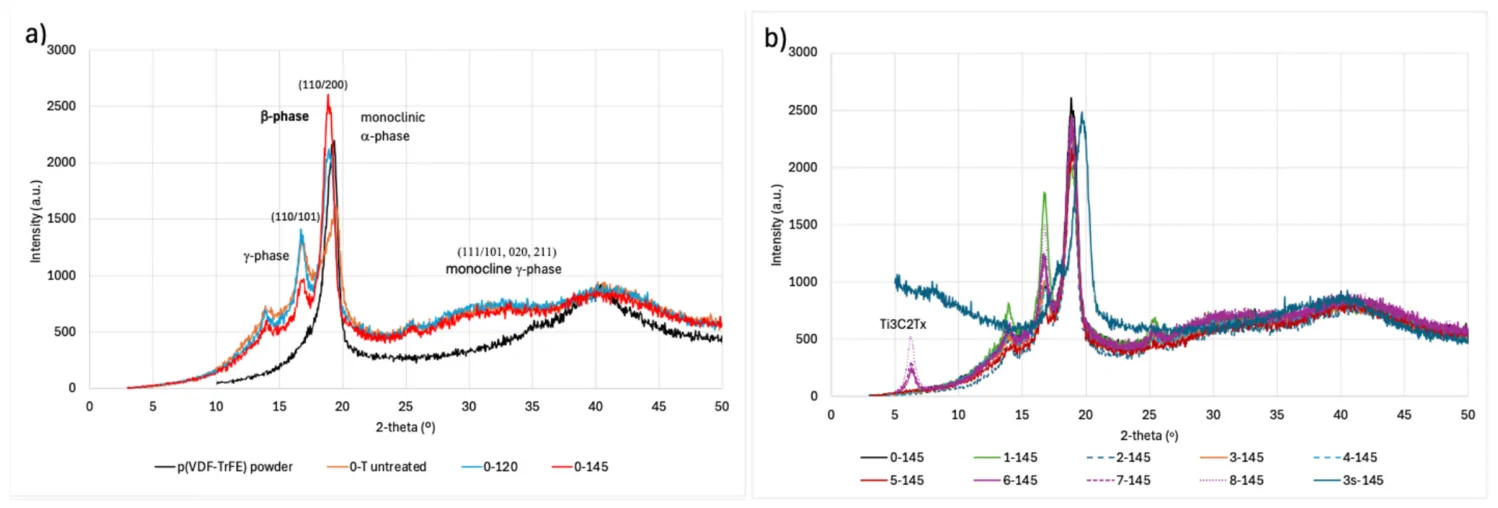
XRD spectra of (**a**) pristine p(VDF-TrFE) films before and after annealing at 120 °C and 145 °C, and (**b**) differently prepared p(VDF-TrFE) film composites as a function of Ti_3_C_2_T_x_ MXene content, annealed at 145 °C, before and after 50% uniaxial elastic deformation (stretching, s) at room temperature.

The crystalline structure and morphology of the p(VDF-TrFE) were evaluated from the XRD patterns of the powder and the films, as shown in Figure 4. The p(VDF-TrFE) powder is mainly in α-phase (non-polar), as indicated by the presence of one intense peak at a 2*θ* value of ~18.9° associated with the (100) and (020) crystal planes, and another weak and broad peak at ~40°, indicating a highly preferred (021) plane orientation of the p(VDF-TrFE) chains. The narrowing and sharp increase in the characteristic diffraction peak of the crystalline paraelectric β-phase at 2*θ* of ~19.3° attributed to the Bragg diffraction of ortochrombic (110) and (200) crystal phases was evident for the thermally annealed p(VDF-TrFE) film [28], indicating an increase in crystal grain size and a higher degree of crystallinity [29]. In addition, one medium and two smaller peaks appeared at ~16.8°, ~14.6° and ~26.6°, corresponding to diffraction peaks on planes (110/101), (020), and (021) of the monoclinic g- and α-phase crystals, respectively [30]; meanwhile, the non-polar α-phase at ~40° was reduced. At the low concentration of Ti_3_C_2_T_x_ nanosheet addition, no additional peaks were observed for the p(VDF-TrFE) spectra, while, at its higher concentrations (samples from no. 5), a characteristic peak for a larger interplanar distance between well-separated layers of Ti_3_C_2_T_x_ MXene appeared at 2θ of ~6.5°, corresponding to its (002) plane structure [31,32]. The composite films still exhibited the same peaks for p(VDF-TrFE) regardless of the MXene addition, and maintained a well-defined β-phase crystal form, but the peak intensity decreased with increasing the MXene concentration. That means the addition of MXene did not affect the crystal phase, but rather decreased the crystal size and degree of crystallinity of p(VDF-TrFE). The intensity of the g- and α-phase peaks in the composite samples is, consequently, slightly enhanced, and, interestingly, was higher for the lower amount of MXene addition. That may be attributed to improvements in both the crystal orientation and crystallinity induced by the MXene, which is consistent with the FTIR analysis.

### 3.3. Effect of MXene Addition on the Films’ Mechanical Properties

**Figure 5:**
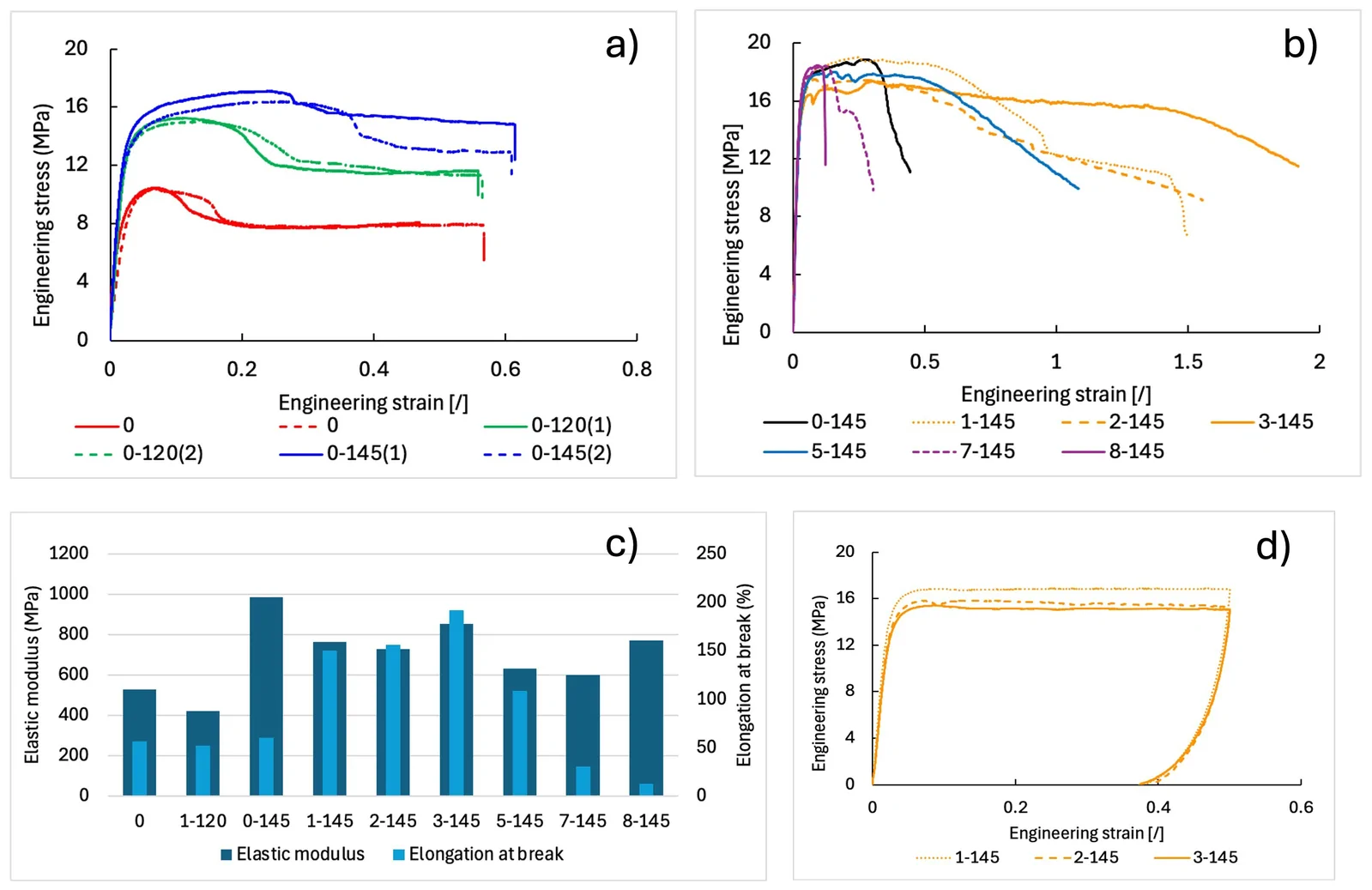
The stress–strain curves of p(VDF-TrFE) film (**a**) after different thermal annealing, and (**b**) different MXene addition, annealed at 145 °C. (**c**) Mechanical properties determined from the average engineering stress–strain curves. (**d**) Representative loading–unloading engineering stress–strain curves of the selected composite film (No. 3) obtained during a single cyclic tensile test up to 50% engineering strain. Average curves are presented for three independent specimens tested under identical conditions.

The uniaxial tensile testing of the films was performed to identify the effect of the addition of MXene to p(VDF-TrFE) by measuring the tensile strength, and providing the Young’s modulus, yield strength, ultimate tensile strength and elongation at break (Table S1). The representative engineering stress–strain curves of the pristine P(VDF-TrFE) films subjected to different thermal treatments are presented in Figure 5a. For all the processing conditions the duplicate tensile tests showed very similar stress–strain responses, confirming the excellent repeatability of the experimental procedure. Only minor differences were observed between the replicate measurements, which were within the expected specimen-to-specimen variability, and can be attributed to slight differences in film thickness and local microstructural heterogeneity. The untreated film exhibited the lowest mechanical performance, reaching a maximum engineering stress of ~10 MPa, followed by an early decrease in stress and a relatively short post-yield deformation region. Thermal treatment influenced the tensile behaviour of the film significantly. Film treated at 120 °C exhibited substantially higher engineering stress throughout the deformation process, reaching ~15 MPa before entering a gradual post-yield softening region, demonstrating enhanced mechanical stability during tensile loading. A further improvement in both the load-bearing capacity and the deformability of the film was observed when treated at 145 °C, reaching ~16.5 MPa of stress over a broad strain interval before gradual stress relaxation occurred, indicating a significant improvement in its ductility. The enhanced mechanical performance resulting from thermal annealing of the film is attributed to thermally induced microstructure rearrangements (i.e., increased crystallinity), which improve the stress transfer during tensile deformation. However, because p(VDF-TrFE) polymer crystallises into the highly rigid, polar β-phase, it is generally more brittle than the thermally untreated, non-polar α-phase film. This structural rearrangement increases the material’s stiffness, which reduces the elongation at break down toward the ~60% range significantly.

All the composite films (containing MXene) annealed at 145 °C exhibited the characteristic tensile response of p(VDF-TrFE), consisting of an initial linear elastic region followed by yielding, strain softening and final fracture (Figure 5b). The representative stress–strain curves reveal that the MXene incorporation affected primarily the post-yield deformation behaviour rather than the initial elastic response. However, low MXene concentrations extended the deformation region significantly after yielding while maintaining a high load-bearing capacity. The ultimate tensile strength remained within a relatively narrow range (16.21–19.03 MPa), while the yield strength varied only slightly (15.76–18.27 MPa) (Table S1), indicating that MXene incorporation had only a limited influence on the onset of plastic deformation and the maximum tensile strength. In contrast, the elongation at break was affected strongly by the MXene addition. The pristine p(VDF-TrFE) film exhibited the highest Young’s modulus (~983 MPa), but only moderate ductility, with an elongation at break of ~60%, indicating a relatively stiff and less deformable material. The incorporation of low concentrations of MXene (samples No. 1–3) improved the films’ deformability markedly. Among all the investigated compositions, the film containing the lowest MXene addition (No. 1) exhibited the highest ultimate tensile strength (~19 MPa), whereas the film with slightly higher MXene content (No. 3) exhibited the greatest elastic modulus (~851 MPa) and elongation at break (~192%), demonstrating the highest ductility. This behaviour is consistent with the representative stress–strain curves, which exhibited a pronounced extension of the post-yield deformation region for these formulations. Although the elastic modulus of this sample was lower than that of the pristine film, it remained higher than that of most other composite films, indicating that substantial improvements in ductility were achieved without a pronounced loss of stiffness. Such an enhanced mechanical performance at low MXene addition is attributed to the formation of a smoother, more homogeneous and defect-free composite microstructure, as already evidenced by the SEM images (Figure 1), which promotes more efficient stress transfer and delays crack initiation during tensile loading, which is highly important for the application. Increasing the MXene content further (samples Nos. 7 and 8) resulted in a pronounced deterioration of the mechanical performance, i.e., an earlier stress drop after yielding, and fracture occurred at significantly lower strains, with the elongation at break decreasing to ~30.5% and ~12.3%, respectively. This behaviour indicates a transition from ductile to brittle fracture, and is most likely associated with the formation of structural heterogeneities and local stress concentration sites caused by excessive MXene loading, which promote premature crack initiation and propagation. Similar behaviour has also been reported by [32].

The films (No. 3) exhibiting the most favourable elastic response were selected for cyclic tensile behaviour (i.e., subjected to a single loading–unloading cycle up to an engineering strain of 50%), in order to evaluate their mechanical stability and recovery under such a tensile loading (50% of elastic deformation), and to investigate whether it affected the films’ crystallinity, dielectric and piezoelectric properties. the 50% elastic deformation Prior to testing, electrodes were deposited onto both surfaces of each of three specimens and remained attached throughout the testing. All the samples exhibited highly reproducible mechanical responses during both the loading and unloading stages (Figure 5d), demonstrating excellent repeatability of the cyclic tensile behaviour. During loading the engineering stress increased rapidly within the initial elastic region and reached a nearly constant plateau between 15 and 17 MPa, which was maintained until the maximum engineering strain of 50% was reached. Minor differences in the plateau stress can be attributed to slight variations in film thickness or local microstructural heterogeneity. During unloading, all the specimens followed a nonlinear recovery path characteristic of viscoelastic polymeric materials. The unloading curves also exhibited excellent agreement, indicating that the applied deformation did not induce premature failure or significant mechanical degradation. No evidence of stress-induced cracking, irreversible damage, or mechanical instability was observed, confirming that the selected film possesses sufficient mechanical integrity to withstand repeated tensile deformation without structural failure. Consequently, any changes observed in the dielectric or piezoelectric properties after mechanical loading can be attributed primarily to deformation-induced microstructural or dipolar rearrangements, rather than to irreversible mechanical damage of the material.

### 3.4. Effect of MXene Addition on Film Dielectric and Piezoelectric Properties

**Figure 6:**
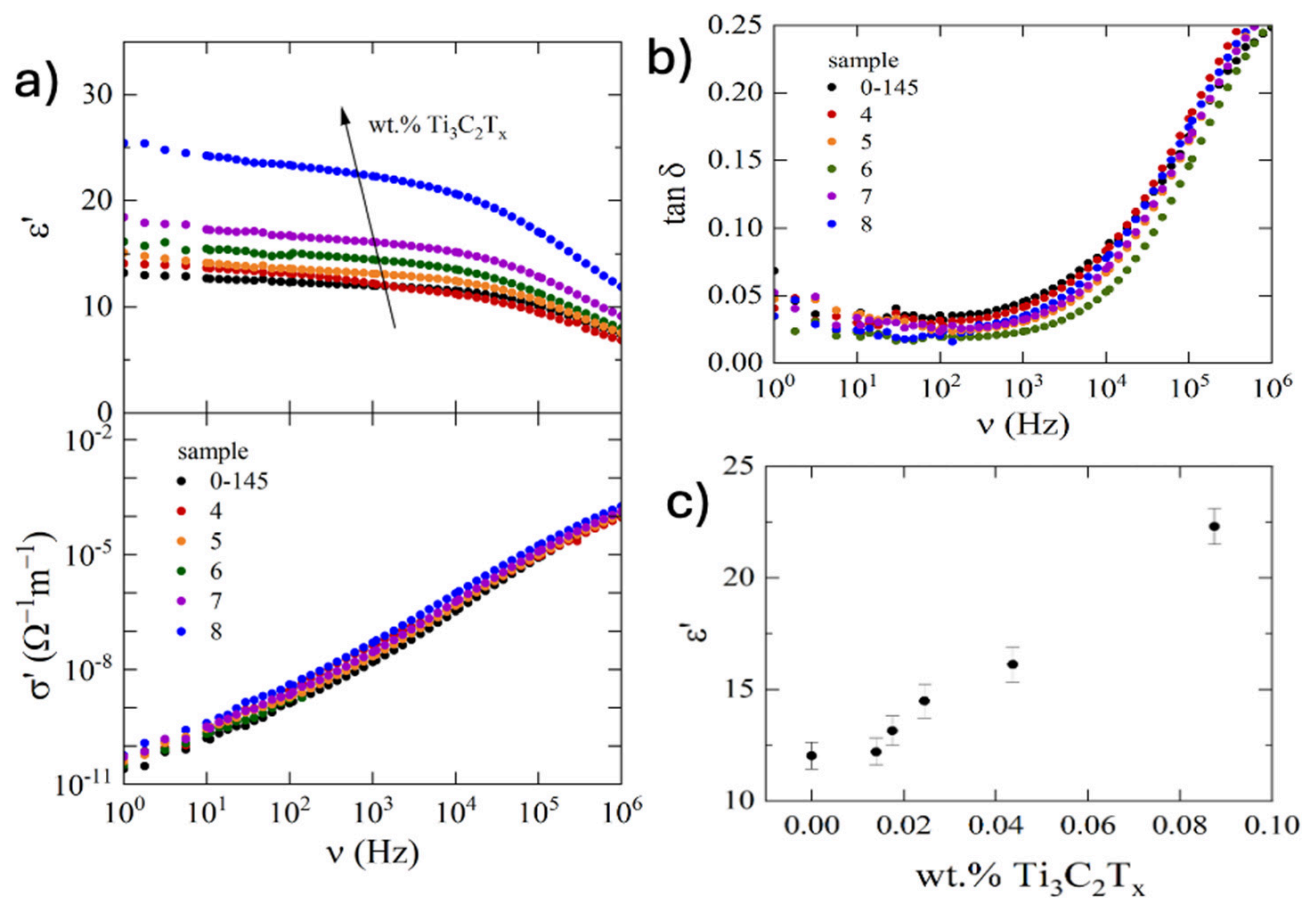
Frequency dependences of the (**a**) dielectric permittivity and electrical conductivity, and (**b**) dielectric loss, detected at room temperature in p(VDF-TrFE) films with and without various MXene content, and annealed at 145 °C. (**c**) Evolution of the room temperature dielectric permittivity at the measurement frequency of 1 kHz as a function of the MXene content. The error bars represent the variation between the measurements of several samples with identical MXene content. Due to clarity, the response of samples 1–3, which is almost identical to the response of samples 0–145 and 4, has been omitted in both graphs.

Figure 6a shows the frequency dependence of the dielectric permittivity and the electrical conductivity of pure p(VDF-TrFE) film and its blends with MXene after annealing at 145 °C. The annealing allows most crystallisation of the p(VDF-TrFE) polymer, which corresponds to a change in the polymer conformation, i.e., the transition between the ferroelectric and the paraelectric phase, yielding a higher harvesting output performance (up to ~18 pC/N) piezoresponse (Table 1), following previous research [27,33]. The composite films show higher dielectric permittivity values than those of the pure film; the evolution of ε’ as a function of the MXene content is shown in Figure 6c. Figure 6b reveals that at very low MXene concentrations, the tan(δ) values decrease because the increase in the dielectric constant is more pronounced than the increase in electrical conductivity. At higher MXene concentrations, however, the increase in electrical conductivity becomes increasingly dominant, causing the dielectric loss to increase with increasing MXene content. This behaviour is generally observed in metal–insulator composites. Although the frequency-dependences of ε’, σ’, and tan(δ) resemble the response of a composite with randomly distributed metallic regions within a dielectric matrix, the increase in ε’ is not only due to enhanced Maxwell–Wagner interfacial polarisation at the p(VDF-TrFE) interfaces, but also due to the preferred orientation of the polymer chains, especially at the crystalline–MXene interfaces [30], which gives rise to a higher dielectric permittivity value at minimal MXene addition (samples nos. 1–3). The 2D nanosheets act as nucleating agents for the electroactive β-phase through surface-interacting functional groups that stabilise the all-trans chain conformation, thereby enlarging the lamellar (β-phase) dimensions and facilitating domain alignment by forming stronger electrostatic intermolecular interactions [15,34,35]. Micro-large 2D substrates are also highly prone to inducing transcrystallization. Because the nucleation density along the broad microlarge MXene surface is extraordinarily high, the polymer crystals cannot grow horizontally into traditional spherical structures (spherulites), but are forced to grow tightly packed and perpendicular to the MXene plane. This yields an anisotropic, highly ordered crystalline layer extending directly outward from the MXene sheets. This also leads to a remarkable improvement in the mechanical properties of the composite films, thereby increasing the dielectric permittivity and piezoelectric coefficient to ~24 pC/N after 50% tensile elongation. However, further increase in MXene changed the crystallinity (Figure 3 and Figure 4), due to higher micronucleation caused by the filler, and impacted the response by film instability, i.e., its breaking during polishing. Relatively larger (4–8 µm) flakes create localised regions that restrict the mobility of the surrounding p(VDF-TrFE) polymer chains. Such a restriction leads to high nucleation density across the entire surface of the flakes, and at excessively high concentrations, they re-stack or self-agglomerate, which can further inhibit polymer chain movement and reduce overall crystallinity. Distributing the strain more effectively without compromising the piezoelectric domains or electromechanical conversion efficiency is highly important in flexible elastomeric sensors [18] and nanogenerators [17].

The dielectric permittivity enhancement of MXene-p(VDF-TrFE) composite films was also shown to be related strongly to the charge accumulation at the surfaces between the MXene flakes and the polymer matrix under an externally applied electric field. It was shown in a previous study that the ratio of permittivity to the loss factor of the large flake composite (ca. 4.5 μm) is superior (10^5^ near the percolation limit of 15.3 wt% MXene loading) to that of the small flake (ca. 1.5 μm) composite (10^4^ near the percolation limit of 16.8 wt%) [36,37]. It should, however, be stressed that based on the experiments performed in the present study, the individual contributions of Maxwell–Wagner interfacial polarisation and dipolar polarisation associated with chain orientation cannot be quantitatively distinguished.

## 4. Conclusions

Micro-sized 2D Ti_3_C_2_T_x_ MXene nanosheets were used as nucleating agents, both to increase and align the electroactive (β-phase formation) crystallinity of p(VDF-TrFE) film anisotropically, and consequently its piezo-response, as well as to improve the stress transfer under mechanical deformation of solvent-casted and thermally annealed composite films. The results demonstrated that thermal treatment enhances (and distributes homogeneously) β-phase formation, piezo-response and the tensile performance of pristine p(VDF-TrFE) significantly. A clear correlation was observed between the film’s elastic modulus and the amount of MXene added. When prepared with a small addition of MXene, the film exhibited much higher macroscopic flexibility, with an ultimate elongation at break increasing from ~60% to ~192% under tensile testing, indicating substantial improvements in ductility without a pronounced loss of stiffness. This is attributed to a more homogeneous composite microstructure that promotes efficient stress transfer and delays crack initiation under tensile loading, which is highly important for the final application. Further addition of MXene reduced ductility significantly and promoted brittle fracture. Such composite films also exhibited higher dielectric permittivity values than the pure film. Although the frequency-dependent dielectric response resembles that of a composite containing randomly distributed MXene regions within a dielectric matrix, the increase in dielectric permittivity is attributed not only to enhanced Maxwell–Wagner interfacial polarisation, but also to the preferred orientation of the polymer chains, as suggested by—albeit limited—indirect FTIR spectroscopy analysis. Although the study does not include direct evidence to distinguish between these two mechanisms, it can be assumed that 2D nanosheet fillers act as nucleating agents for the electroactive β-phase through surface-interacting functional groups, thus also stabilise the all-trans chain conformation. This also improves piezo-response and significantly enhances the mechanical stability of such a composite film, thereby enhancing both the dielectric permittivity and the piezoelectric coefficient, even after 50% tensile deformation (i.e., elongation).

## Figures and Tables

**Table 1:** Preparation and labelling of the films prepared from 7 wt% p(VDF-TrFE) and various MXene additions, and their piezoelectric response d33 before and after 50% uniaxial elastic deformation (stretching) at room temperature (RT).

Sample No.	0.05 wt% MXene (mL)	d33 (pC/N) as Prepared	d33 (pC/N) After 50% of RT Elongation
0	0	0	/
0–120 °C		14	
0–140 °C		17	
1	0.7	18	22
2	1.4	18	22
3	2.1	19	24
4	2.8	* 15	/
5	3.5	* 10	/
6	4.9	**	/
7	8.75	**	/
8	17.5	**	/

* Time-reduced piezo response. ** Sample broke during polishing.

## Data Availability

The original contributions presented in the study are included in the article/Supplementary Materials. For additional information, please contact the corresponding author.

## Supplementary Materials

The following supporting information can be downloaded at https://www.mdpi.com/article/10.3390/polym18202488/s1. Figure S1: Morphology and elemental structure of MXene, as obtained by SEM-EDX imaging. Figure S2: Photos of differently prepared films. Figure S3: FTIR spectra of (a) pure and (b) differently prepared p(VDF-TrFE) film composites as a function of Ti_3_C_2_T_x_ MXene content and film annealing temperature. Table S1: Mechanical parameters determined from the average engineering stress–strain curves of p(VDF-TrFE) films containing different MXene concentrations.

## Abbreviations

The following abbreviations are used in this manuscript:

p(VDF-TrFE)	poly(vinylidene fluoride-co-trifluoroethylene)
MEK	Methyl ethyl ketone
