# Supplementary material for: Ti3C2Tx MXene-Supported Anisotropic Crystallisation of Solvent-Casted p(VDF-TrFE) Films for Improved Elastic Deformation and Piezoelectric Response
Source: Polymers (Basel). 2026 Oct 12;18(20):2488. doi: 10.3390/polym18202488 (PMC13708805; doi:10.3390/polym18202488)
Supplement: Supplementary file 1 [file polymers-18-02488-s001.zip › polymers-4586489-supplementary.pdf]

$Ti_3C_2T_x$ MXene-supported anisotropic crystallisation of solvent-casted p(VDF-TrFE) films for improved elastic deformation and piezoelectric response

Vanja Kokol [1,*], Branko Nečemer [1], Eugenio Gibertini [2], Vid Bobnar [3]

[1] University of Maribor, Faculty of Mechanical Engineering, Maribor, Slovenia, vanja.kokol@um.si
[2] Politechnic University of Milan, Department of Chemistry, Milan, Italy, eugenio.gibertini@polimi.it
[3] Jožef Stefan Institute, Department of Condensed Matter Physics, Ljubljana, Slovenia, vid.bobnar@ijs.si
* Correspondence: Vanja.kokol@um.si; Tel.: +386 2 220 7896

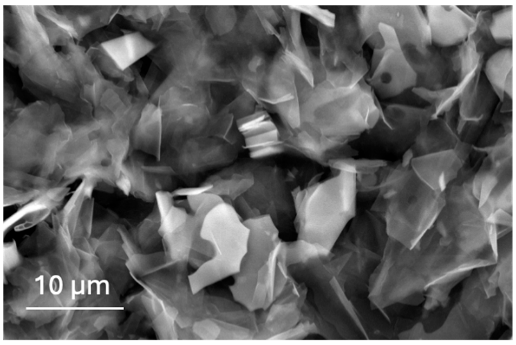

Ti (wt%)	C (wt%)	O (wt%)	F (wt%)	Cl (wt%)
39.92±14	39.8±16	15.50±8	7.05±3	1±0.5

Figure S1. Morphology and elemental structure of MXene, as obtained by SEM-EDX imaging.

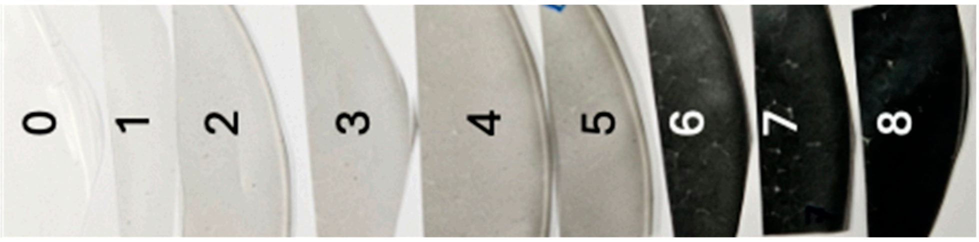

Figure S2. Photos of differently prepared films.

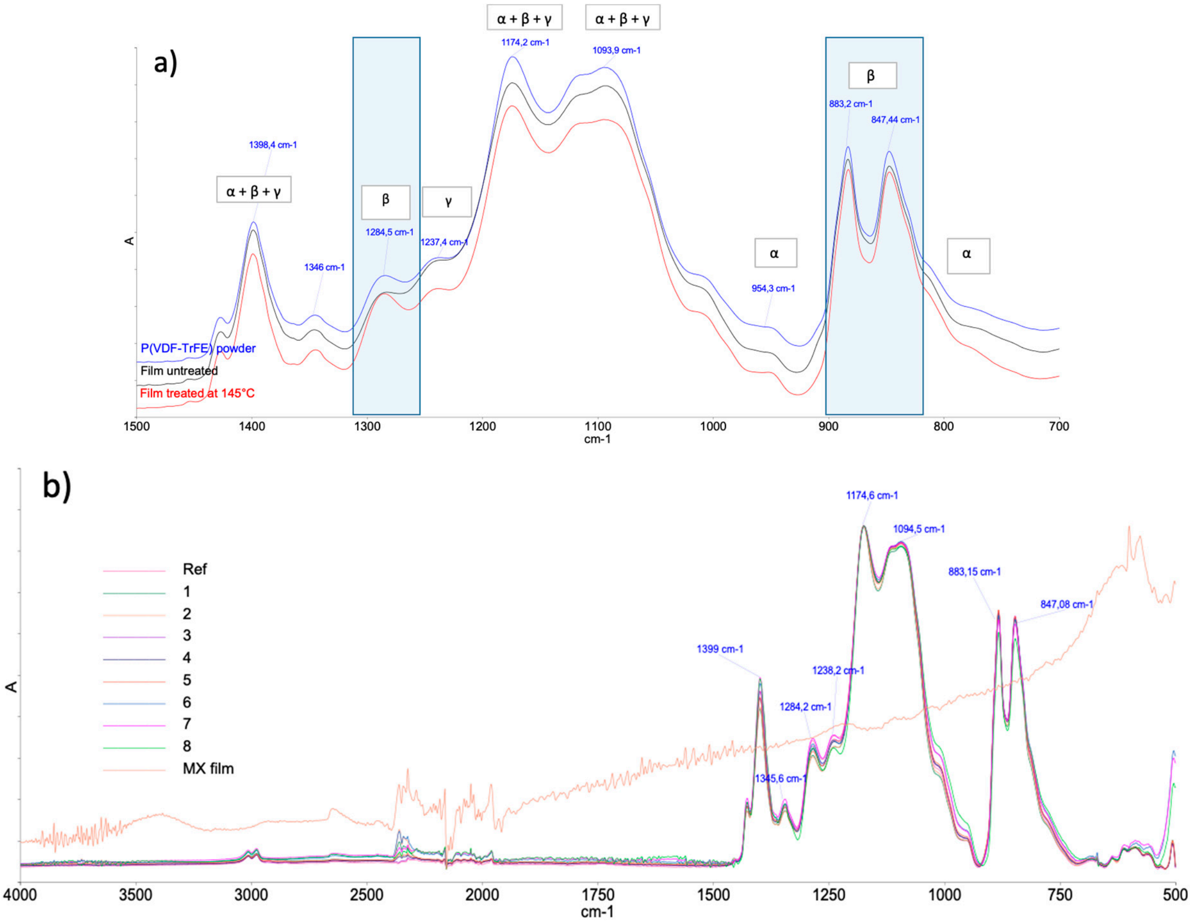

Figure S3. FTIR spectra of a) pure and b) differently prepared p(VDF-TrFE) film composites as a function of $Ti_3C_2T_x$ MXene content and film annealing temperature.

Table S1. Mechanical parameters determined from the average engineering stress–strain curves of p(VDF-TrFE) films containing different MXene concentrations.

Sample	Elastic modulus (MPa)	Yield strength (MPa)	Tensile strength (MPa)	Elongation at break (%)
0	527.66	9.68	15.13	56.15
0-120	420.9	7.48	10.46	51.84
0-145	983.05	15.76	16.73	60.09
1-145	762.32	18.27	19.03	149.90
2-145	728.47	17.50	17.50	155.60
3-145	851.84	16.12	16.21	191.80
5-145	629.88	17.80	17.99	108.50
7-145	598.93	17.70	18.42	30.50
8-145	768.34	18.27	18.44	12.30
